# HIV/AIDS treatment funding system to support the people affected by HIV/AIDS in Surakarta, Indonesia

**DOI:** 10.1080/17290376.2020.1858946

**Published:** 2021-01-28

**Authors:** Argyo Demartoto, Bhisma Murti, Siti Zunariyah

**Affiliations:** a Department of Sociology, Universitas Sebelas Maret; b Department of Public Health, Universitas Sebelas Maret

**Keywords:** ARV, funding, health insurance, PLWHA

## Abstract

People Living with HIV/AIDS (PLWHA's) quality of life (QoL) is determined by the lifetime treatment sustainability. Republic of Indonesia Minister of Health's Decree Number 328 of 2003 stated that government subsidies the PLWHA's medication and treatment, despite not covering entire medication and treatment cost. The objective of research was to analyse the cost assumed by PLWHA in accessing HIV/AIDS treatment service in Surakarta, Indonesia. The target group in this case study was PLWHAs, and related stakeholders of medical treatment in one of Public Health Centers and a Public Hospital in Surakarta; AIDS Commission of Surakarta City; Solo Plus Peer Support Group and AIDS-Care NGO selected purposively. Data collection was carried out using observation, in-depth interview, and documentation. Method and data source triangulations were used to validate data that was then analysed using Grossman's Demand for Health Capital theory. The result of research showed that the sources of HIV/AIDS treatment cost were self-income, Social Insurance Administration Organization (BPJS) fund and Local Government subsidy. Admission and physican services are given for free to PLWHA because it has been paid by BPJS Fund or has been subsidied by Local Government. Otherwise, they should pay registration cost of IDR 50,000, in Public Hospital and IDR 75,000 in Private Hospital. Physician service costs IDR 50,000–IDR 200,000. VCT Counsellor costs IDR 35,000-IDR 150,000. Non-Subsidy ARV costs IDR 687,000. 1 bottle containing 60 TB meningitis drug capsules costs IDR 145,000 for 10–20 d use and maximally IDR 210,000, while herpes drug costs IDR 295,000. CD4 examination costs IDR 126,000-IDR 297,000, RNA Viral load IDR 1,275,000–IDR 1,471,000, Haematology IDR 60,000-IRD 90,000, Cholesterol and triglyceride IDR 100,000-IDR 250,000, and SGOT/SGPT IDR 100,000–IDR 200,000. There is monthly non-medical cost the patient should spend, including transportation cost to go to health centre, and food, beverage, and newspaper cost while waiting for the service. BPJS fund and local government subsidy relieved health economic burden of PLWHAs, so that the average HIV/AIDS treatment cost in PLWHAs was relatively low, less than 10% of expense. National Insurance System including BPJS fund and local government subsidy as the answer to the integration of HIV/AIDS treatment funding management into national insurance system had provided PLWHA a funding access involving prevention, care, support, and treatment, and mitigated the effect despite less optimum.

## Introduction

Some countries develop policy aiming to expand pre-paid system and to reduce the dependency on out-of-pocket system as quickly as possible by developing broader and more just funding system through tax, social health insurance, and dual healthcare system (Apanga, Punguyire, & Adjei, 2012; Creese, Floyd, Alban, & Guinness, 2002; Galarraga et al., 2011; Ganesh & Rampersad, 2018; Goldie et al., 2006; Lamontagne, Over, & Stover, 2019; Moon et al., 2008; Pérez-Molina, Martínez, Blasco, Arribas, & Gatell, 2019). In Indonesia, it is mentioned in Republic of Indonesia Health Minister's Regulation Number 21 of 2013 about HIV/AIDS overcoming. Central and local governments obligatorily allocate budget to fund HIV/AIDS overcoming, to ensure the availability of drug and health equipment necessary, including care, support, and treatment (CST) of People Living with HIV/AIDS (PLWHA) assumed by the state. Every health insurance organiser obligatorily assumes some or entire medication and treatment costs of PLWHAs insured. It is also confirmed with Republic of Indonesia Health Minister's Regulation Number 28 of 2014 about Guidelines of National Health Insurance Program implementation. The funding sources of AIDS overcoming in Indonesia come from public through State Income and Expense Budget and Local Income and Expense Budget minus social health insurance for poor people (*Jamkesmas*) and social health insurance provided by provincial or district government (*Jamkesda*); multilateral international donor (Global Fund/GF, UN Agencies and EU) and bilateral (Australia Government through DFAT and American Government through USAID); and private such as Indonesian Business Coalition on AIDS (IBCA). Some international NGOs support the HIV/AIDS coping programme in Indonesia particularly for both the most at risk populations (MARPs), such as Injecting Drug Users (IDU), Men who have Sex with Men (MSM), and Sex Workers (SW) and their clients; PLWHAs and the public. They are Family Health International (FHI), the Ford Foundation, Asia Pacific Network for PLHIV (APN+), HIVOS, UNAIDS, Treat Asia and etc. In addition, many foundations and NGOs operate in HIV/AIDS field, including Indonesia AIDS Coalition (IAC), NGO forum caring about AIDS, GWL-INA, Ardhanary Institute, Women Journal Foundation, National Commission for Women, and Indonesian Commission for Child Protection at national level; NGO for Children with HIV/AIDS, NGO of Harm Reduction, NGO based Women Human Right at regional level; and Foundation for MSM community and Solo Transsexual Association at local level.

Everyone including PLWHA is entitled to access a high quality and affordable universal healthcare service (Bemelmans et al., 2010; WHO, 2016a; WHO, UNAIDS and UNICEF, 2008). Several factors affecting PLWHA's quality of life are, among others: coinfection, availability, ARV, compliance with ARV, CD4 number, social support, occupation, gender, stigma, and depression rate (Atkins et al., 2010; Fatiregun, Mofolorunsho, & Osagbemi, 2009; Folasire, Irabor, & Folasire, 2012; Loutfy et al., 2015; Odili, Ikhurionan, Usifoh, & Oparah, 2011; Parslow, Jorm, Christensen, Jacomb, & Rodgers, 2004; Razavi et al., 2012). Summary of global HIV epidemic 2016 shows that 36.7 million people are now estimated to be living with HIV and until mid-2017 there are 20.9 million people receiving antiretroviral treatment (WHO, 2016b). Cumulatively, there have been 232.323 HIV, 86.780 AIDS and 14.608 deaths in Indonesia in the period of April 1987–December 2016. A recent scientometric analysis identified that there is a knowledge gap in economic context for particular settings and sub populations (Tran et al., 2019). Central Java is on the 13th position out of 34 provinces in Indonesia with 16,867 HIV and 6444 AIDS cases (Ditjen & Kemenkes, 2016). Meanwhile, cumulatively there have been 596 HIV/AIDS cases consisting of 246 HIV, 350 AIDS and 144 deaths in Surakarta in the period of 2005–December 2017. It is estimated that 80% of PLWHA receive Antiretroviral (ARV). Republic of Indonesia Health Ministry's Decree Number 328 of 2003 about National Formulary states that ARV is subsidised by government through providing hospital and Public Health Center to which HIV/AIDS treatment is referred. Considering the Republic of Indonesia Minister of Health's data per December 2018, the availability of ARV is guaranteed by government and it can be utilised for free. ARV service can be access in Hospitals and Public Health Center in 34 provinces, 227 regencies/cities. Totally there have been 896 ARV services consisting of service that can initiate ARV therapy and satellite service. Social support from family and closest environment is desirable for PLWHA to keep having spirit and to continuetheir medication.

Social, economic, and politic change dynamics and some challenges arising in funding HIV/AIDS overcoming in Indonesia because GF as main contributor beginning to reduce its donation, inadequate government budget and business world's inadequate concern make AIDS overcoming as a target of SDGs not interpreted yet optimally into Standard Minimum Service. Meanwhile, HIV/AIDS cases in Indonesia increase continuously while HIV/AIDS funding allocation in Indonesia is only 42% belonging to low category compared with that in Philippines (52%) or Malaysia (97%) (UNAIDS, 2017).

People's access to high-quality universal healthcare service begins to improve through the activation of Social Insurance System. However, HIV/AIDS treatment is the lifetime one, so that treatment cost burden and sustainability contribute to PLWHA's quality of life. The guideline of National Health Insurance (Indonesian: *Jaminan Kesehatan Nasional* or JKN) for HIV-AIDS and IMS in Healthcare Center states that there are some fees for administration/admission, medical action, cotrimoxazole prevention therapy (CPT), Isoniazid Preventive Therapy (IPT), ARV drug, Laboratory Test, CD4 Examination, Virology (Viral Load) Examination, and Opportunistic Infection Treatment and/or Side Effect of ARV on People with HIV/AIDS. Admission or registration fee should be paid by the patients per visit or consultation (Ditjen & Kemenkes, 2015). HIV/AIDS treatment cost increases when PLWHA develops opportunistic infection (OI) due to damaged immune system such as tuberculosis (TBC), chronic diarrhoea, oropharingeal candidiasis, generalised dermatitis, and persistent generalised limphadenopathy (Greener, 2006; Onah, Pharm, & Pharm, 2016; Rosen, Ketlhapile, Sanne, & DeSilva, 2007; Walensky et al., 2006). In some countries with low and medium incomes, it is estimated that 5.2 millions PLWHAs have received ART and treatment continuation is highly dependent on the availability of programme budget and treatment cost per patient annually (Galarraga et al., 2011). OI drug cost is also assumed by providing drug for accompanying diseases. So, in addition to getting ARV, PLWHAs get antibiotics with type and number corresponding to prevent resistance (Vogenberg, 2001).

In health economics, there is demand for medical care as the derivation of demand for health as basically an individual needs health and for that reason, healthcare service is needed. Individual health is affected by individual's income. Healthcare service is a derivative demand functioning to be input to produce health. PLWHAs fulfil their health actively, spend time to improve their health, and use healthcare service all at once. Thus, health is considered as consumptive material and investment all at once, as it is durable and not depreciated immediately (Grossman, 1972).

Drug has economic and expertise symbols. Good health and quality of life are affected not only by the presence of pharmacy but also occupation and income (Duncan, 2017; Grossman, 1972). It affects PLWHA's accessibility to HIV/AIDS treatment service, while travelling distance and time do not matter to avoid stigma and discrimination (Handayani, Herman, Mujiati, & Masitoh, 2017; Phelps, 2003; Zweifel, Breyer, & Kifmann, 2009) but no travelling difficulty was the predictor of higher adherence to HIV treatment (Mai et al., 2018).

Health investment is expensive because it takes not only money (fund) but also time. From limited resource [fund and time], everyone will decide the optimum health the patients can afford. PLWHA can predict the effect of the changing healthcare service tariff and healthcare product, job opportunity and salary, and technology in health industry (Fuchs, 1996; Grossman, 1972). This research aimed to study and to show the cost the PLWHA should assume in accessing HIV/AIDS treatment service in Surakarta Indonesia. This study's findings are expected to increase the knowledge on HIV/AIDS, how it should be treated, and to be the consideration material to the policy makers related to the treatment of PLWHAs.

## Material and methods

### Study design and area

This qualitative with case study was conducted in Surakarta, Central Java, Indonesia. The study was important to do in Surakarta because the number of HIV/AIDS cases in Surakarta occupied the 2nd rank in Central Java and it really becomes an iceberg phenomenon with the ever increasing number. There have been 309, 386, 477, 589 HIV/AIDS cases in the periods of 2014–2017 respectively. This research was conducted from July to September 2017. Considering the number of case in relation to HIV/AIDS funding system, it is important to conduct this research to study and to indicate the cost the PLWHA should assume in access HIV/AIDS treatment service in Surakarta Indonesia.

### Data source

Unit of analysis used in this research consisted of individual, family, and institution related to HIV/AIDS treatment funding system in Surakarta including key informant, main informant, and supporting informant. Health workers in medical record, admission, pharmacy, and financial departments in one of Public Health Center and Public Hospital in Surakarta; Solo Plus Peer Support Group (PSG), Program Manager of Surakarta City's AIDS Commission and NGOs caring about AIDS served as key informant because the institution knows the identity and representation of PLWHAs in Surakarta. Meanwhile, health workers catering to HIV/AIDS patients in hospitals and Public Health Centers in Surakarta know data and information related to treatment funding system for HIV/AIDS patients in corresponding hospital and Public Health Center. PLWHAs coming from various risks, age, sex, occupation, income, marital status, OI, dan fund sources served as main informant. Meanwhile, the supporting informant included PLWHAs’ family or People Affected by HIV/AIDS (PABHA), staff of NGOs caring about AIDS and health workers in one of Hospital and Public Health Centers in Surakarta catering to HIV/AIDS patients. It is because the family, in this case the participants of Social Insurance Administration Organization (Indonesian: *Badan Penyelenggara Jaminan Sosial* or BPJS) and Surakarta City Public Health AID (Indonesian: *Bantuan Kesehatan Masyarakat Kota Surakarta* or BKMKS) Program, is affected directly by the treatment funding system for its family members with PLWHA status. As known, the participants of BPJS and BKMKS are family and/or individuals corresponding to the provision.

Primary data was obtained directly from 18 informants consisting of 4 key informants: Program Manager of AIDS Commission, Chairperson of Solo Plus PSG, Chairperson of HIV/AIDS Work Group and physician of VCT Clinic in one of Public Hospitals in Surakarta, and VCT counsellor in one of Public Health Centers in Surakarta. The main informants were 7 PLWHAs becoming the members of Solo Plus PSG consisting of 5 PLWHAs with high risk of being infected with HIV/AIDS including a FSW, a transsexual, a gay, an IDU, and a high risk man or HRM, and 2 PLWHAs coming from ordinary people including a housewife infected with HIV, and a driver of cross-provinces bus. Meanwhile, 7 supporting informants consisted of 2 PABHAs including a gay's elder brother/sister and B4's father, and 2 health workers including C3 (registration and medical staffs in one of Public Health Centers in Surakarta) and C4 (pharmacy staff in one of Public Hospitals in Surakarta), C5 (Program manager of Women Human Right-based NGO), C6 (Program Director of NGO's Harm Reduction) and C7 (Chairperson of NGO for Children with HIV/AIDS). Meanwhile, secondary data derived from document relevant to AIDS treatment funding in Surakarta Indonesia (Creswell & Poth, 2016).

### Sampling technique

The sampling technique used in this study was non probability purposive sampling and snowball sampling technique. The author determined who would become the sample and the size of sample; the sample was taken gradually with increasing number of informants (Coyne, 1997; Morse, 2000; Sandelowski, 1995; Shaheen, Pradhan, & Ranajee, 2018; Suri, 2011). The author identified an individual considered as knowledgeable on the problem studied, and then he could determine the next informant. This technique was used as it corresponds to the objective of research, to analyse the HIV/AIDS treatment funding system for PABHA in Surakarta. The author selected 18 informants representing PLWHAs with varying risk, age, sex, occupation, income, marital status, OI, and fund sources, PABHAs and institutions related to HIV/AIDS treatment funding system in Surakarta.

### Technique of collecting data

The author went straightly to the research location to observe all activities conducted by informant related to the HIV/AIDS treatment funding system. The author observed type of medical service and laboratory accessed by informant, transportation used to go to healthcare service centre, and food and beverage consumed, including newspaper bought during waiting for the service. Through the observation, it can be found how the fund is spent by informant related to HIV/AIDS funding system. The author also conducted non-structured interview because he felt ‘having no knowledge on what he has not known’. The interview was conducted in informal structured way with open-ended question leading to the information depth and to explore the subject of research's view on HIV/AIDS treatment funding system to explore information more in-depth. Interview guide was organised flexibly with the paraphrasable open-ended questions corresponding to the need and condition during interview. The author developed different interview guides for different status and position of informants related to the data and information on HIV/AIDS treatment funding system. Content validity and face validity had been tested on June 15, 2017 at 09.00 am local time on the observation and interview guide in the research entitled HIV/AIDS Treatment Funding System for PLWHA in Surakarta, Central Java, Indonesia and had been approved by Research and Community Service Institution of Universitas Sebelas Maret (Indonesian: *Lembaga Penelitian dan Pengabdian kepada Masyarakat Universitas Sebelas Maret* or LPPM of UNS) (Patton, 2002).

In addition, the author made citations from document existing in the research site, sorted and selected them corresponding to the objective of research and then review them. As known, a large number of facts and data relevant to HIV/AIDS treatment funding system were stored in documentary materials including letters, diary, report, photograph, and etc. This main data was borderless either temporally or spatially, thereby giving the author the opportunity of finding out anything having ever occurred. In detail, documentary material was divided into some categories: autobiography, personal letter, diary, memorial, clipping, government and private document, data in the server and flashdisk, data stored in website, and etc. The author classified official document related to HIV/AIDS treatment funding system into two categories: internal and external documents, produced by one of Public Health Centers and Public Hospitals in Surakarta; Solo Plus PSG, Surakarta City's AIDS Commission, and NGOs caring about AIDS in the last five years. Internal document can be note including memo, announcement, instruction, an institution's rule, enacted system, meeting minute or leader's decision, and etc. External documentation can be information materials including magazine, newspaper, bulletin, statement, and etc. The data cited were then sorted and selected, and reviewed corresponding to the objective of research (Yin, 2017).

### Data analysis

Typology analysis technique was conducted by sorting and arranging data, and then reading entire description, extracting the informant's significant statements and grouping them by research objective and integrating them into narrative description (Flick, von Kardoff, & Steinke, 2004; Yin, 2017). The type of data analysis used in this research was holistic one, analysing the case holistically or in the term of the interrelationship between cases found and experienced by PLWHAs, PABHAs, and institution related to HIV/AIDS treatment Funding System. It is an analysis specific for and limited to HIV/AIDS treatment funding system for PLWHA in Surakarta. In this case, the author employed pattern matching logic by comparing the patterns based on empirical data with the predicted one (or with some alternative predictions) before the research period. In this research, if these two patterns contain similarity, the result can confirm the internal validity of case study conducted (Yin, 2017). Thereafter, the author made explanation aiming to analyse the case study data found in the field.

### Data validity and reliability

Data validation was conducted using method and data source triangulations through verifying the result of interview with one informant and another, including observing informant behaviour and studying document relevant to research object (Miles, Huberman, & Saldana, 2014). To verify the extensive data, the author employed triangulation and member check. The author searched for information centres related directly to HIV/AIDS treatment funding system for PABHA in Surakarta, in this case PLWHAs and related institutions. Thus, data validation can be verified using member check and data comparison (Jack & Baxter, 2008; Stake, 1995).

### Ethic

To ensure the informants’ confidentiality, the research was completed with informed consent document related to data obtained. To protect the PLWHA as the subject of research, all data and information obtained from the informant are safeguarded for their confidentiality and only used for research purpose (Orb, Eisenhauer, & Wynaden, 2001). Before filling in the informed consent, all informants were informed about the objective of research, the confidentiality of identity and information given. Meanwhile, when there is a statement generating discomfort, the author would cease data collection and allow the informant to withdraw from the research without any risk. It was confirmed with ethical clearance from the Health Research Ethics Committee of Dr. Moewardi General Hospital and Faculty of Medicine of Universitas Sebelas Maret Surakarta Number: 1.022/VI/HREC/2017 dated June 27, 2017. The author had fulfilled the informants’ five rights: self-determination, privacy and dignity, confidentiality, the right to equal management, the right to get protection from discomfort and loss. Consensual decision making or informed consent approach was conducted to fulfil those five rights (American Nurses Association, 2014; Andorno, 2007; Walker, 2007).

## Results

### Characteristics of informant

There were 18 informants employed in this study: 4 key informants (A1-A4), 7 main informants (B1-B7), and 7 Supporting Informants (C1-C7). Characteristics of main informants (PLWHA) in this research can be seen in Table 1. PLWHAs studied are 20–45 years old. They are the members of a peer support group in Solo and some of them are active in many HIV/AIDS prevention and overcoming activities conducted by Surakarta AIDS Commission, Solo Plus PSG and People caring about AIDS in respective areas. PLWHAs’ income ranges between IDR 0 and IDR 5,250,000. Four PLWHAs have gotten married and 4 others have not yet. Five (5) PLWHAs belong to the group with high risk of being infected with HIV/AIDS: FSW (B1), Gay (B2), transsexual (B3), IDU (B4) and HRM (B5). In addition, there is a housewife infected with HIV (B6). B6 was infected with HIV from his HIV-positive husband and her husband has passed away due to AIDS now. B6 have HIV-infected baby due perinatal transmission. Meanwhile B7 is a HIV-positive driver with high mobility and severe smoking habit. HIV/AIDS level of all PLWHAs studied is on the 1^st^ stage. There are 4 PLWHAs developing opportunistic infections including: B1 developing herpes zoster, B3 developing oral candidiasis, and B4 and B5 developing TB meningitis. PLWHAs have their health examined on their own initiative, supported by family, PSG, and NGOs caring about AIDS.

**Table 1.** Characteristics of main informants.

No	Name	Age [Year]	Sex	Occupation	Income (Rupiah)	Marital Status	Risk Factor	Stage	IO	Fund Source
1	B1	36	Female	FSW	2.500.000,	Married	Heterosexual	I	Herpes Zoster	BPJS
2	B2	32	Male	Hotel Manager	5.250.000	Not married	Homosexual	I	-	Self fund
3	B3	28	Transsexual	Sex worker	2.000.000	Not married	Homosexual	I	Oral candidiasis	BPJS
4	B4	20	Male	College student	-	Not married	IDU	I	TB Meningitis	BPJS
5	B5	45	Male	Entrepreneur	4.750.000	Married	HRM	I	TB Meningitis	Self fund
6	B6	34	Female	Housewife	-	Married	Heterosexual	I	-	BKMKS
7	B7	40	Male	Driver	2.750.000	Married	Heterosexual	I	-	BPJS

In this study, the supporting informant was selected, i.e. PABHAs, consisting of PLWHAs’ family, staff of NGOs caring about AIDS and health workers catering to HIV/AIDS patients in one of Public Hospitals and Public Health Centers in Surakarta. PLWHAs’ family because the ones becoming the participants of BPJS are individuals and or family corresponding to BPJS’ requirement. Meanwhile, Surakarta people becoming the participants of BKMKS are those coming from poor family with Surakarta Identity Card. They are affected directly by treatment funding system for family members with PLWHA status. Meanwhile, the health workers in one of Public Hospitals in Surakarta and some Public Health Centers catering to HIV/AIDS patients in Surakarta were selected to be supporting informant because they know data and information related to treatment funding system for HIV/AIDS patients in hospital and Public Health Center where they work.

Seven (7) supporting informants were: C1 (B1's sister), C2 (B4's father), C3 (staff of admission and medical record department in one of Public Health Centers in Surakarta), C4 (staff of Pharmacy Department in one of Public Hospitals in Surakarta), C5 (Program Manager of Women Human Right-based NGO), C6 (Program Director of NGO of Harm Reduction), and C7 (Head of NGO for Children with HIV/AIDS). It can be seen in Table 2.

**Table 2.** Characteristic of supporting informant.

No.	Informant	Sex	Age (Year)	Education Level	Position	HIV Status
1.	C 1	Female	27	Senior High School	B1's sister	-
2.	C 2	Male	54	Senior High School	B4's father	-
3.	C 3	Female	31	Undergraduate	Staff of admission and medical record Department in one of Public Health Centersin Surakarta	-
4.	C 4	Female	35	Undergraduate	Staff of Pharmacy Department in one of Public Hospitals in Surakarta	-
5.	C 5	Female	36	Bachelor	Program Manager of Women Human Right-based NGO	-
6.	C 6	Male	46	Bachelor	Program Director of NGO's Harm Reduction	-
7.	C 7	Male	53	Senior High School	Head of NGO for Children with HIV/AIDS	-

### HIV/AIDS treatment funding

**Figure 1.**
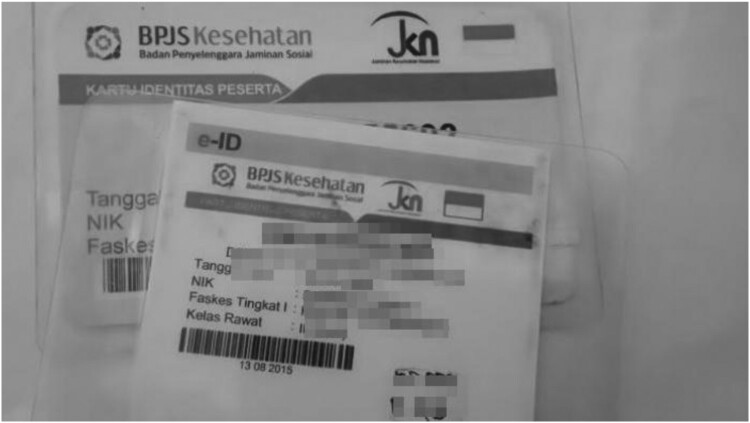
BPJS card that can cover treatment fee for HIV/AIDS and OI without complication. Source: Processed Primary Data, October 2017.

PLWHA's QoL is determined by internal and external dimensions. One of them is CST through outpatient care. Considering the research, the fund source of outpatient PLWHA treatment comes from self-income, BPJS, Local Government Subsidy and etc, such as family, friend, NGO, PSG, and church's help. PLWHA having BPJS treatment insurance and local government subsidy have zero (0)-treatment fund, except when such the facility is not used. Figure 1 shows card that can cover treatment fee for HIV/AIDS and OI without complication.

**Figure 2.**
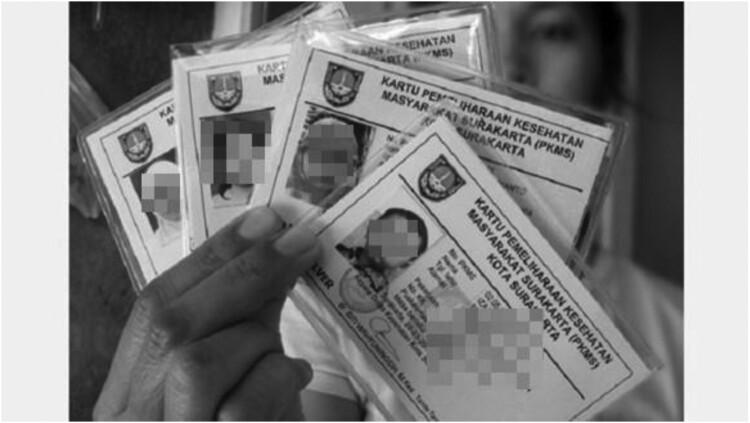
BKMKS card covering HIV/AIDS treatment fund particularly for poor people in Surakarta. Source: Processed Primary Data, October 2017.

HIV/AIDS treatment fund can derive from individual and family's income source. Family will assume the treatment fee for its family members including wife, husband, or child with HIV/AIDS and not-working status. Meanwhile, unmarried but independent PLWHAs will pay their medication themselves. It is related to JKN belonging to PLWHAs. The fund source of HIV/AIDS and OI and non-complication OI treatments for PLWHAs (B1, B3, B4 and B7) comes from BPJS fund covering HIV/AIDS treatment cost. Meanwhile B2 uses his own fund for treating HIV/AIDS. Because of his sexual orientation, B2 often gets negative stigma and discriminative treatment. However, B2 devises to be the participant of BPJS to relieve treatment cost when he is sick. To be the participant of BPJS, all members of family included in family card should apply for it. B5 is PLWHA becoming the participant of BPJS but not using the facility because of complicated administrative requirements. So some stages should be passed through from family physician/proximate Public Health Center to Public Hospital and length of service time, so that B5 assumes his HIV/AIDS treatment cost himself. Ironically, as the participant of BPJS, B5 spends his own money for HIV/AIDS and OI treatment such as registration, physician examination, ARV and OI drug, and laboratory examination. Meanwhile, B6 and her baby infected with HIV come from poor family so that they get aid from Surakarta City Government based on Surakarta Mayor's Regulation No.21 A of 2017 about Local Health Insurance for Poor People through Surakarta City Public Health AID programme. Solo's poor people who have not followed other health insurance programmes such as BPJS and private insurance can register themselves to be the participants of BKMKS in the local *Kelurahan* Office rather than coming to Surakarta City's Health Service. The requirements they should fulfil to register are e-Demographic Identity Number, copy of Family Card, and Child Incentive Card. Another requirement is that the one registering is the resident who has stayed permanently in Solo for three successive years. The number of participants is not limited but the fund use is limited to maximally IDR 5 millions per people annually. Surakarta City Government has allocated budget of IDR 11.7 billions to BKMKS purpose. The fund is calculated from an assumption that everyone gets fund of IDR 5 millions per year. Figure 2 shows BKMKS card covering HIV/AIDS treatment fund particularly for poor people in Surakarta.

### Medical cost

HIV/AIDS and OI treatment cost includes medical cost covering registration, medical service, counsellor/physician service, drug, and laboratory examination. Medical cost of PLWHA is calculated based on the cost spent by PLWHA. It is shown in Table 3. Registration and physician service costs worth 0 (zero) for patient using BPJS or getting Local Government subsidy, while PLWHA using their own fund should pay registration costs of IDR 50,000 in Public Hospital and IDR 75,000 in Private Hospital. Physician service costs minimally IDR 50,000 and maximally IDR 200,000. VCT Counsellor costs minimally IDR 35,000 and maximally IDR 150,000. Non-Subsidy ARV costs IDR 687,000. Corresponding to guidelines of National Health Insurance, BPJS covers all types of diseases including those belonging to medical indication such as non-complication OI. It can be found in B1 (Herpes zoster), B3 (oral candidiasis), B4 and B5 (TB meningitis). However, sometimes B1 buys herpes drug herself costing IDR 295,000 and B5 always buys a bottle containing 60 TB meningitis drug capsules costing IDR 145,000 and maximally IDR 210,000 for 10–20 d use. Additionally, B1, B2, B3, and B5 always buy multivitamin to maintain their body stamina. They spend about IDR 70,000 – IDR 100,000 monthly. Meanwhile, B6 should buy formula milk monthly to fulfil her HIV-infected baby's nutrition, because she as PLWHA may not breastfeed her baby exclusively. A 900g-box of formula milk costs IDR 319,000.

**Table 3.** Medical cost.

No	Name	Health Facilities	Fund Source	Registration (IDR)	Service		Drug		
					Physician (IDR)	Counsellor (IDR)	ARV (IDR)	IO (IDR)	Personalised Prescription (IDR)
1	B1	Public Hospital	BPJS	0	0	0	0	295,000	0
2	B2	Private Hospital	Self-fund	75,000	200,000	150,000	687,000	0	20,000
3	B3	Public Hospital	BPJS	0	0	0	0	0	0
4	B4	Public Health Center	BPJS	0	0	0	0	0	0
5	B5	Public Hospital	Self-fund	50,000	50,000	35,000	300,000	145,000	10,000
6	B6	Public Hospital	BKMKS	0	0	0	0	0	0
7	B7	Public Hospital	BKMKS	0	0	0	0	0	0

B1, B2, B3, B4 and B5 argued that majority PLWHAs are lower-middle class people. Thus government ensures PLWHAs to get 1^st^-line ARV or first Nucleoside analogue Reverse Transcriptase Inhibitors (NRTIs) for free from Public Health Centers or hospital. This non-subsidy 1^st^-line ARV is very expensive and even, so is the 2nd-line ARV. C4 argues that when PLWHAs do not comply with the 1^st^-line ARV consumption, the tendency to replace this drug with the 2nd-line ARV is higher. Therefore, PLWHAs should comply with ARV consumption in order not to spend extra money on obtaining the 2nd-line ARV.

A3 said that ARV treatment is a lifetime one and should be conducted routinely and regularly with periodical control or evaluation. If PLWHAs discontinue their medication, they will worryingly develop drug resistance, treatment failure or clinical condition exacerbation. ARV should be administered in combination with at least 3 drug regimens to deal with a variety of mutations occurring in the virus during treatment process. Without combination, HIV can develop its mutation so that this virus becomes drug-resistant. Generally, Tenofovir and Zidovudin are main choices for 1^st^-line ARV. If tenovofir cannot be used, it can be replaced with zidovudin, and vice versa. If both of them cannot be used, another choice is Stavudin. In certain population, for example HIV patients with hepatitis B, Tenofovir can be the main choice.

A3 stated that Efavirenze-type of ARV has been produced by one of drug factories in Indonesia, distributed to hospitals, and is expectedly capable of discontinuing dependency on ARV drug imported so far. A3 added that Efavirenz is the fourth type of ARV that can be produced by drug factory, following lamivudine, zidovudine, and nevirapine. Other types and the 2nd-line ARV are still imported from India, but they are often distributed lately to hospitals. The price of 3^rd^-line ARV is so expensive that government should spend about IDR 14 millions monthly for a PLWHA; therefore PLWHA can get it only by buying it with their self fund.

B1, B3, B4 and B6 said that health insurance system provided through BPJS or local government subsidy relieves PLWHAs’ burden in accessing healthcare service. B2 suggested that HIV/AIDS treatment in one of Private Hospitals in Surakarta needs additional cost of IDR 10,000 – IDR 20,000 for personalised prescription.

### Laboratory test cost

In addition to registration cost, according to C1, C2, C3 and C4, the cost the PLWHA should be paid frequently is laboratory examination cost. Because some hospitals and Public Health Centers have not had HIV/AIDS examination laboratory facilities, the examination is conducted in private clinic laboratory in their own fund. Laboratory examination costs IDR 100,000 – IDR 1,471,000. A3 and A4 stated that supporting examination is consistent with potential contraindication or side effect. There are two important primary screenings to evaluate HIV/AIDS treatment: CD4 and viral load. Despite no clinical symptom, PLWHAs should undertake viral load examination once in 12 months. CD4 should be examined in HIV diagnosis before starting, after starting, during undertaking and in the case of ARV therapy clinical failure. HbsAG is an important supporting examination during HIV diagnosis. Meanwhile, viral load is examined in the case of immunological or clinical failure. CD4 should be examined periodically to ascertain the treatment response. A2, C3, C4, C5, C6 and C7 argued that the largest laboratory cost is used for Viral Load, CD4, Rontgen, and liver function examination. However, B1, B2, B4 and B6 spend no fund for laboratory examination because of Surakarta City Government's policy supported by Global Fund-AIDS Tuberculosis and Malaria [GF-ATM] manifested into the NGO-caring about AIDS programme in Surakarta.

B6 and B7 knew that government subsidies several healthcare facilities to overcome AIDS, e.g. VCT in Public Health Centers and Local General Hospital with free service and administration cost of IDR 5000– IDR 50,000 only. They get counselling service and CD4 test for free. A3 said that one of Hospitals in Surakarta provides pre-ART test service for free encompassing complete blood, liver function test, renal function test, Rontgen, and CD4 test. A series of tests is used to determine whether or not there is a disease in the body and CD4 test functions to measure the prior number of CD4 before ARV therapy.

PLWHAs, according to B3, should also be disciplined in financial matters. Many PLWHA are absent in their health examination because they have no money. Moreover when there is no occupation and they are still dependent on parents, like B4. Some examinations take much money, e.g. CD4 examination important to find out the body immunity system of PLWHAs conducted routinely once in 3 or 6 months as necessary. This examination can be done in private laboratory with specified facilities. In Surakarta, several public Hospitals to which HIV/AIDS patients are referred have had such facilities with low examination cost, but the facilities are limited in number. B3 undertook CD4 examination in laboratory (private clinic) costing IDR 297,000, while B5 did so in public hospital's laboratory costing IDR 126,000. Then, the examination of HIV RNA or viral load test is a test to measure HIV virus number in blood indicating whether or not ARV therapy controls virus. Similarly, not all hospitals have CD4 examination facilities; thus B3 undertook examination in private laboratory with very expensive cost. This examination is conducted in the beginning of ARV therapy, and then once in a year costing IDR 1,471,000, while B5 undertook viral load test in private hospital's laboratory costing IDR 1,275,000.

Haematology examination, according to A3, is the one intended to find out the condition of blood and its components and to determine the quality of health. Moreover, as PLWHA, any health effect can occur, so that it is important to monitor it. Haematology examination can be conducted in Public Health Center, private hospital and clinic with affordable cost. A4 stated that not only PLWHAs but also everyone living and caring about his/her health condition needs this examination. Haematology examination should be conducted once a month or once in 3 months. In Public Health Center and hospital it costs very low about IDR 60,000, but it can cost IDR 90,000 in private laboratory.

A3 stated that several types of ARV can affect cholesterol and triglyceride conditions of PLWHAs’ body, so that they should undertake cholesterol and triglyceride examination at least once in 3 months. Just like haematology, this examination is provided in many healthcare service centres costing IDR 100,000 – IDR 250,000. C5 and C6 suggested that Serum Glutamic Oxaloacetic Transaminase (SGOT)/Serum Glutamic Piruvic Transaminase (SGPT) or Alanin aminotransferase (ALT) can be undertaken in all healthcare service centres costing IDR 50.000-Rp. 100.000, respectively, so that total SGOT/SGPT examination costs is IDR 100,000 – IDR 200,000. It is shown in Table 4.

**Table 4.** Laboratory examination cost.

No	Name	Health Facilities	Fund Source	Registration Cost (IDR)	Laboratory Examination Cost				
					CD4/3-6 months (IDR)	RNA Viral load/12 months (IDR)	Haematology/3 months (IDR)	Cholesterol and triglyceride 3 months (IDR)	SGOT/SGPT/3 months (IDR)
1	B1	Public Hospital	BPJS	0	0	0	0	0	0
2	B2	Private Hospital	Self fund	75,000	297,000	1,471,000	90,000	250,000	200,000
3	B3	Public Hospital	BPJS	0	0	0	0	0	0
4	B4	Public Health Center	BPJS	0	0	0	0	0	0
5	B5	Public Hospital	Self fund	50,000	126,000	1,275,000	60,000	100,000	100,000
6	B6	Public Hospital	BKMKS	0	0	0	0	0	0
7	B7	Public Hospital	BKMKS	0	0	0	0	0	0

### Non medical cost

There is monthly non-medical cost the PLWHAs should spend, including transportation cost to go to healthcare centre, that for buying food, beverage, and newspaper while waiting for the service. It can be seen in Table 5. In C1 and C2's opinion, it is conducted because HIV/AIDS treatment service sometimes takes long time. All of the PLWHAs as the informants in this research always spend money for buying fuel and for paying public transportation fee. Transportation cost was dependent on the distance of house to healthcare service and the type of transportation mode used. Transportation of PLWHA costs minimally IDR 10,000 and maximally IDR 100,000. Food purchasing costs minimally IDR 2,500 and maximally IDR 10,000, mineral water costs IDR 2,500 and bottle tea maximally IDR 5,000, while newspaper costs IDR 3,500-5,000. Non-medical cost is minimally IDR 50,000 and maximally IDR 100,000.

**Table 5.** Non medical cost.

No	Name	Distance of house to healthcare service centre (Km)	Transportation		Other Costs		
			Type	Cost (IDR)	Food (IDR)	Beverage (IDR)	Magazine/ Newspaper (IDR)
1	B1	5	Motor	10,000	5000	2500	0
2	B2	12	Car	100,000	0	2500	5000
3	B3	2	Motor	10,000	5000	5000	3500
4	B4	4	Motor	10,000	10.000	2500	0
5	B5	12	Car	10,000	5000	5000	0
6	B6	2.5	Public transportation	20,000	7000	5000	0
7	B7	2.5	Public transportation	0	0	0	0

### Sustainable comprehensive service for overcoming HIV/AIDS

Surakarta City government's commitment to overcome AIDS, TB, and malaria has been mentioned clearly in its vision: the realisation of Surakarta as an independent, advanced, and prosperous cultural city. To achieve the vision, Surakarta city determines to be free of AIDS, TB and Malaria through the mission of Realising Surakarta people who are *Waras, Wasis*, *Wareg, Mapan*, and *Papan* (healthy, educated, sated, well-established, and having shelter or house). *Waras* (healthy) mission is translated into realising a physically and spiritually healthy community in healthy living environment. Therefore, Surakarta aims to improve healthcare service accessibility and quality, to take promotive and preventive measures related to physical and spiritual public health and to improve the quality of urban living environment. Sustainable Comprehensive Service for overcoming HIV/AIDS consists of main element of healthcare service (primary, secondary and tertiary) from both government and private. Meanwhile coordination is conducted between such elements as Local AIDS Commission, Local Apparatus Work Unit and people such as NGO, community organisation, religion organisation, and PSG.

The Surakarta City's Local Action Plan for HIV/AIDS Prevention and Coping of 2017–2022 explains that HIV/AIDS prevention and coping programme in Surakarta City involves prevention programme with supporting budget of IDR 220,540,000, Medication and treatment programme and support for PLWHA of IDR. 171,400,000, – and Supporting Programme of IDR 248,910,000. Then, Surakarta City's AIDS Commission develops a strategy of coping with HIV/AIDS in Surakarta during 2017–2022 involving: reinforcing the HIV/AIDS programme leadership and management, improving access to high-quality service, controlling risk factor, improving partnership, and improving community independency in HIV/AIDS prevention and overcoming. Meanwhile, the CST programme involves: improving medication, treatment, and laboratory service facilities for STD, HIV, and AIDS cases, opening VCT clinic for high-risk group and patient, ARV drug availability to ensure the sustainable treatment for PLWHAs, drug availability for opportunistic infection, drug availability for STD, and Case management. To deal with HIV/AIDS epidemic, Surakarta City's Government has reformed some areas particularly in allocating the supporting budget to health and social welfare service sector. Therefore, in the operational implementation of HIV/AIDS coping work, the Surakarta's AIDS commission optimises 7 work groups: Prevention and Outreach, CST, Management Reinforcement, Harm Reduction, Empowerment, HIV prevention through monitoring and evaluation, and HIV and TB work groups. The strong commitment of all community members to assuming that HIV/AIDS epidemic is the shared responsibility of government and community is expected to suppress HIV/AIDS spread in Surakarta.

**Figure 3.**
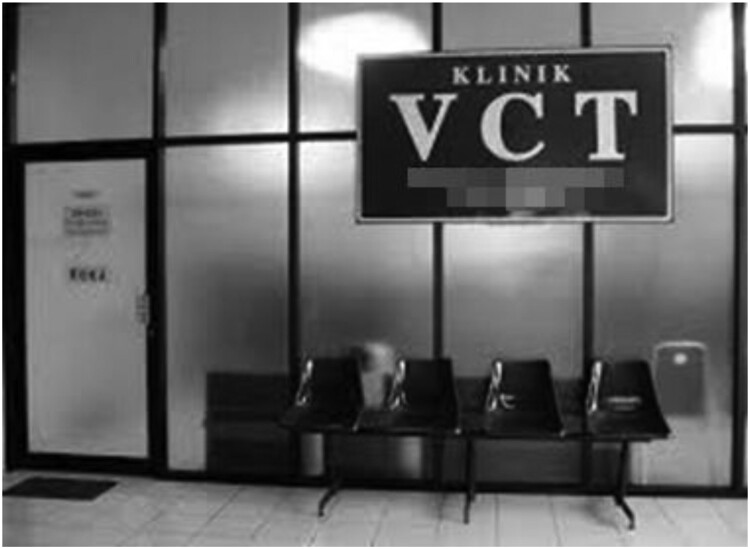
VCT Clinic Facilities in one of Public Hospital in Surakarta. Source: Processed Primary Data, October 2017.

A1 said that continuum of care including promotive, preventive, curative, and rehabilitative services can decrease HIV prevalence. Primary healthcare service includes non-specialist, promotive and preventive, examination, treatment, medical consultation, non-specialist medical action, drug and consumable medical materials, and first-level laboratory diagnostic supporting examination, while outpatient service belongs to Public Health Center's capitation. A2 mentioned hospitals and Public Health Centers providing ARV service for free in Surakarta including some of Public Hospitals, Private Hospitals, Public Pulmonary Health Center of Surakarta and 17 Public Health Centers. Public Health Centers providing services for Sexually Transmitted Infections (STIs), HIV counselling and test, HIV counselling and test on Health Workers’ Initiative are Public Health Center Banyuanyar and Setabelan. Public Health Center Sangkrah provides services for STIs, HIV Counselling and Test, HIV counselling and test on Health Workers’ Initiative, and CST, while Public Health Center Pajang, Penumping, Purwosari, Jayengan, Gajahan, Purwodiningratan, Ngoresan, Sibela, Pucangsawit, Nusukan and Gilingan provide HIV counselling and test on Health Workers’ Initiative only. Meanwhile, Public Health Center Manahan provides STIs, HIV Counselling and Test, HIV counselling and test on Health Workers’ Initiative, methadone, sterile injection, and CST. Figure 3 shows VCT Clinic in one of Public Hospitals in Surakarta.

NGOs and community organisations caring about HIV AIDS in Surakarta provide sustainable (promotive, preventive, curative, and rehabilitative) comprehensive service related to HIV/AIDS prevention and coping. For example, NGO for women and children, NGO for Harm Reduction, MSM comumunity conducting HIV/AIDS prevention and coping in MSM community, religious organisations and then Family Welfare Program, Youth Forum, and etc. Women Human Right-based NGO, Child Protection, and Family Planning and Public Health Centers in Surakarta serving as the executor conduct IVA, VCT, and STD examination (tests) in *Kelurahan* Danukusuman office. Before taking the test, Women Human Right-based NGO team educates the participants about the importance of IVA, VCT, and STD tests, and how to get ARV, and examination technique to detect STD and HIV/AIDS earlier. The participants were very enthusiastic and the cadres actively invited the people to undertake the examination. The result of examination is so confidential that patients and medical staffs handling them only may know it.

In addition, mass organisation such as Village Commissioner (Indonesian: *Bintara Pembina Desa*) and Public Health Centers in Surakarta monitor the area actively in implementing early detection and the VCT mobile activities in most-at-risk group and the public at *Kelurahan* level in Surakarta City. These activities also involve giving education about HIV/AIDS hazard, how to avoid the disease and to access ARV.

**Figure 4.**
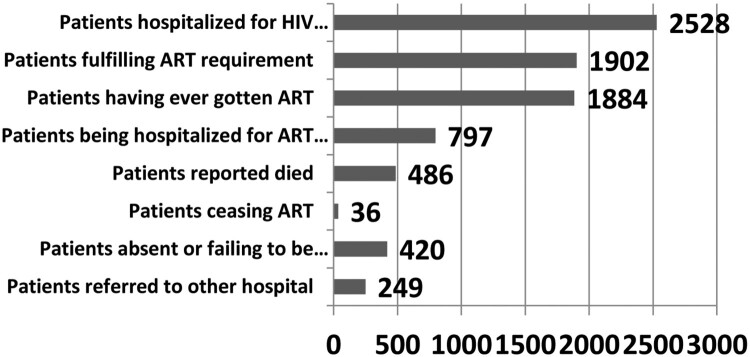
ART Cascade of Surakarta City. Source: AIDS Commission of Surakarta City, December 2017.

Considering the result of data compilation from some healthcare institutions providing HIV/AIDS treatment service in Surakarta, it can be found ART Cascade up to December 2017 shows 2528 patients hospitalised for HIV, 1902 fulfilling ART requirement, 1884 having ever received ART, 797 being hospitalised for ART treatment, 486 reported died, 36 ceasing ART, 420 absent or failing to be followed up, and 249 referred to other hospital. This data is displayed in Figure 4.

## Discussion

HIV/AIDS overcoming enters into a new chapter in Indonesia because social, economic, and political conditions change very rapidly. The sources of HIV/AIDS treatment cost were self-income, BPJS fund and Local Government subsidy. Before 2015, the largest proportion of HIV/AIDS overcoming fund came from GF grant constituting the most important part of total fund for ATM programme. However, the problem of donor funding impacts directly the performance of programme, thereby reducing the GF grant and domestic (State and Local Income and Expense Budgets) and other sources funding as a very important part of exit strategy now. It affects the central and the local governments’ commitment to increasing the fund. HIV/AIDS treatment funding has been allocated from local source, but it is still dependent on budget allocation for health sector including 5% of State Income and Expense Budgets and 10% of Local Income and Expense Budgets beyond salary as mandated by Republic of Indonesia's Law Number 36 of 2009 about Health. Thus, universal coverage is expected to be fulfilled gradually.

Every individual including PLWHA attempts to achieve certain health status by investing and or consuming a number of healthcare products or services corresponding to demand for health capital he/she has (Grossman, 1972). In this case, the product is indicated with drugs consumed by PLWHAs contributing to PLWHA's sustainability. Meanwhile, service is indicated with HIV/AIDS treatment service, support and medication accessed by PLWHA. Income affects the demand for good healthcare service as both health capital and derived demand in the attempt of maintaining certain health level. It can be found that the source of fund for PLWHAs’ ambulatory healthcare service comes from their own income, BPJS, Regional Government subsidy, and etc such as family, friend, NGO, PSG, and Church's helps. And in undertaking the medication, some healthcare service centres are accessed corresponding to PLWHAs’ body condition and need. In relation to some options of public healthcare centres available, the costs spent by PLWHAs are varying dependent on each option. It is because the effective demand for health will occur when consumers have willingness and ability of paying a number of healthcare service type needed (Grossman, 1972).

However, PLWHAs have not been satisfied in the sense of accessing the healthcare service comfortably and easily. Elaborate administration, high cost, spatial gap, discrimination, and other problems, either internally or externally, inhibit the PLWHAs from accessing the healthcare service. To be the members of BPJS, many constraints are faced related to many administrative documents to be completed to get referral to the healthcare service centre according to the disease developed and long time taken to do the process. Thus, PLWHAs use their own fund for HIV/AIDS medication.

Until today, the need for ART in Indonesia, including Surakarta, is still subsidised by Government, thereby it is helpful to PLWHAs. It can be seen from the improvement of PLWHAs’ survival in Surakarta. With the PLWHAs’ higher accessibility to ART drug and their compliance with consuming ART twice a day, their CD4 level improves and virus HIV is undetected (Bacheler et al., 2000). The development of paediatric ART has substantially changed HIV/AIDS perception from very fatal disease to chronic and likely acute one. The availability and the administration of ART therapy and compliance with ARV consumption have evidently reduced HIV/AIDS-related mortality and morbidity rates (Arts & Hazuda D, 2012; Lodi et al., 2011; Oguntibeju, 2012). Factors affecting the need for ART, among others, are ARV drugs chosen resulting in various side effects, physician's devotion including limited time dedicated to counselling and building mutual trust, PLWHAs receiving no ART as well as its advantage and disadvantage, and social background and family unwilling to persuade PLWHA to continue ART (Iacob, Iacob, & Jugulete, 2017; Rathbun, 2017). Viewed from health economics perspective, incompliance with treatment increases treatment cost with high cost of alternate drug and hospitalisation duration (Vogenberg, 2001; Onah et al., 2016). Sufficiently high ARV treatment, particularly when patients encounter virological failure in 1st-line, the 2nd-line therapy is needed with much higher cost. Life expectancy increases by 30% using the 2nd-line ART after the failure of 1^st^-line regimen (Fuchs, 1996; Goldie et al., 2006; Iacob et al., 2017; Scambler, Higgs, & Jones, 2002).

The survival of PLWHAs complying with ART in Surakarta is much better and longer than that of those not complying with consuming ART. Similarly, those without coinfection have better survival or will live longer than those with coinfection (Dworkin, Hanson, Kaplan, Jones, & Ward, 2000; Paterson et al., 2000). Efavirenz is one of 1st-line ARV types used widely, because of its low side effect on liver function, but it makes the users flying and hallucinated thereby affecting PLWHA's QoL. Dealing with such challenges as HIV resistance, side effect of ARV, and hepatitis C, Indonesia devises to improve the procurement of important drugs in limited manner and more affordable price, including the 3^rd^-line ARV for PLWHAs resistant to 1st- and 2nd-line and hepatitis C drugs. Initially, PLWHAs needing can get the 3rd-line ARV for free, but when the need is considerable, government should think of its funding (Boyer, Sarafianos, Arnold, & Hughes, 2001; Dorr et al., 2005; Kirkcaldy, Spiegel, Abdalla, & Erdelmann, 2006). In addition to 3rd-line drug, Indonesia devises to provide Rilpivirine as alternative to Efavirenz. The side effect of drug is a little but its HIV-inhibiting power is larger.

The most efficient way of inhibiting HIV infection development is to use ARV still becoming golden standard to suppress virus number. Nevertheless, multivitamin (vitamins B, C, and E) and selenium supplement consumption has good ability of reducing risk in those who have not undertaken ARV treatment yet. Multivitamin can reinforce immune system, while selenium supplement provide mineral intake suppressing virus growth (Hendricks, Erzen, Wanke, & Tang, 2010; Kaiser et al., 2006; Naeger, Margot, & Miller, 2002).

The newest strategy of overcoming HIV is called pre-exposure prophylaxis approved by WHO. In some countries, this strategy has been evidently beneficial, particularly to a couple one of which is HIV-positive or to those having many sexual partners. Because they are at higher risk, ARV administration will reduce the risk of transmission by 97%. Some requirements should be fulfilled to get preventive benefit: people should not be infected with HIV surely (should be negative-HIV) and their renal and bone condition is healthy. As preventive measure, ARV should be consumed for many years so that it should be ensured that it has minimum side effect. In contrast to ARV therapy that should be consumed 3–4 drugs daily, ARV prevention measure is conducted by consuming one tablet only everyday (Dykes et al., 2001; Fuchs, 1996; Lalezari et al., 2003; Liu et al., 2016; Riddell, Amico, & Mayer, 2018; Rigourd, Ehresmann, Parniak, Ehresmann, & Marquet, 2002).

OI drugs need considerable fund, because not all of them are supported by BPJS or subsidised by Local Government. In addition, most of those developing OI need antibiotic with relatively expensive price. If PLWHAs undertake inappropriate OI treatment, resistance risk will occur and need larger fund (Ditjen & Kemenkes, 2016). Therefore, in addition to HIV drugs, hepatitis C drugs now attract attention as well. It is estimated that 2 millions Indonesian people live with hepatitis. Hepatitis C is not as popular as HIV but can exert serious effect. If left untreated, hepatitis C seizure can make someone developing liver cancer. Hepatitis C treatment is inhibited by limited fund and expensive cost. The combination of interferon and ribavirin can take patient's money up to IDR 30 millions. The attempt of importing a cheaper hepatitis C drug to Indonesia has been successful. Sofosbuvir, an India-made drug that according to some studies successfully suppresses hepatitis C up to 90 percent, could have been consumed by Indonesian people (Agarwal, Bagchi, & Yadav, 2017; Arjun et al., 2017; Berenguer et al., 2009; Mshana et al., 2006; WHO, 2015).

Laboratory examination, in addition to aiming to enforce diagnosis, is also intended to monitor therapy advance. Several public hospitals encounter some constraints with the examination due to unavailable reagent or damaged facilities, so that examination is undertaken in private laboratory or hospital with larger cost. It also occurred in China, where ARV is given for free to PLWHA. However, other components of treatment like Viral Load test is not subsidised by the government. Thus, it becomes financial constraint for PLWHAs to access HIV/AIDS treatment (Moon et al., 2008). Virus detection standard examination conducted using HIV specimen, PCR DNA and RNA assays is very limited and expensive (Lamontagne et al., 2019; Tuller et al., 2010).

Sometimes, average non-medical cost is larger than average medical cost as most treatment fund is assumed by government, but transportation access still becomes a constraint. Healthcare service approach to give PLWHA an access to ARV should be taken into account (Greener, 2006). HIV/AIDS treatment needs at least 6 visits a year among PLWHAs after beginning ARV treatment, so that transportation and waiting time costs taken by PLWHAs and those accompanying them during treatment process should be taken into account (Hipolito et al., 2017; Pérez-Molina et al., 2019). Generally, the fund taken for one-month treatment is still less than 10% of household expenditure. It belongs to small expenditure, but if it is viewed per household, there is a substantial expenditure because more than 60% of fund is used for HIV-infected housewife and her HIV-positive baby. It is used for buying supplement food such as formula milk as she is not allowed to breastfeed her baby exclusively.

Generally, economic condition of patients does not change significantly before and after HIV/AIDS infection because the drug they consume can recover their physical condition into normal so that they can work routinely to meet their economic need. The government and the foreign grant give the drugs for free to PLWHAs; thus they unnecessarily spend their money for buying ARV. It indicates the government and the foreign donator's responsibility and attention to PLWHAs (Ditjen & Kemenkes, 2016; Rosen et al., 2007). However, at household level, it has been very worrying. Some PLWHAs and households tend to be burdened with many problems such as developing various chronic diseases, losing job and income, increased expenditure for health, and reduced saving or other asset. Compared with expenditure level, the average HIV/AIDS treatment cost for PLWHA is relatively low, less than 10% of expenditure, but treatment cost of B6 reaches 60% of her total monthly expenditure.

The heavy economic burden to be assumed and the health expenditure for HIV-infected household members compelled PLWHAs not to work (Arjun et al., 2017; Hipolito et al., 2017; Odili et al., 2011). Total treatment cost affects not only PLWHAs/family but also government and hospital. Sustainable HIV/AIDS treatment funding need special attention and policy from central and local government, both from service and treatment access aspects (Hongoro, Mturi, & Kembo, 2008). To improve the PLWHA's accessibility to cost aspect, government should formulate an integrative HIV/AIDS treatment package including HIV/AIDS medication and treatment fund in any form of health insurance, from HIV examination, consultation with physician, drug (ART), laboratory and treatment in Hospital if necessary according to the clinical condition. This integrative package is expected to cover some types of HIV/AIDS medications and treatments.

In summary, economic condition also affects PLWHA's QoL. The finding of current study confirmed the findings of previous studies that some factors affect PLWHA's QoL: coinfection, availability, ARV, compliance with ARV, CD4 number, social support, occupation, gender, stigma, and depression rate (Atkins et al., 2010; Fatiregun et al., 2009; Folasire et al., 2012; Loutfy et al., 2015; Odili et al., 2011; Parslow et al., 2004; Razavi et al., 2012).

## Conclusions

The sources of HIV/AIDS treatment cost were self-income, Social Insurance Administration Organization (BPJS) fund and Local Government subsidy. There is monthly non-medical cost the patient should spend, including transportation cost to go to health centre, and food, beverage, and newspaper cost while waiting for the service. BPJS fund and local government subsidy relieved health economic burden of PLWHAs, so that the average HIV/AIDS treatment cost in PLWHAs was relatively low, less than 10% of expense. National Insurance System including BPJS fund and local government subsidy as the answer to the integration of HIV/AIDS treatment funding management into national insurance system had provided PLWHA a funding access involving prevention, care, support, and treatment, and mitigated the effect despite less optimum.

PLWHA's accessibility to HIV/AIDS treatment still finds some constraints including elaborate administrative affairs due to government's technocratic policy domination, spatial discrepancy, high cost, and discrimination. PLWHA's accessibility to HIV/AIDS treatment still finds some constraints including elaborate administrative affairs due to government's technocratic policy domination, spatial discrepancy, high cost, and discrimination.

Therefore, both central and local government, particularly Surakarta City Government should develop an efficient bureaucracy for HIV/AIDS treatment procedure, facilitate the need for HIV/AIDS treatment through BPJS or local government's subsidy in the form of an integrative treatment package, improve the quantity and the variety of healthcare institution catering to HIV/AIDS problems supported with competent human resources and adequate facilities. In addition, it is important to minimise negative stigma and discrimination against PLWHAs and to treat them equally to non-PLWHA patients.
